# Effects of nurse-led motivational interviewing of patients with chronic musculoskeletal pain in preparation of rehabilitation treatment (PREPARE) on societal participation, attendance level, and cost-effectiveness: study protocol for a randomized controlled trial

**DOI:** 10.1186/1745-6215-14-90

**Published:** 2013-04-02

**Authors:** Vera-Christina Mertens, Mariëlle E J B Goossens, Jeanine A Verbunt, Albere J Köke, Rob J E M Smeets

**Affiliations:** 1 Department of Rehabilitation Medicine, Maastricht University, School for Public Health and Primary Care (CAPHRI), P.O. Box 616, Maastricht, MD, 6200, The Netherlands; 2 Adelante Centre of Expertise in Rehabilitation and Audiology, P.O. Box 88, Hoensbroek, AB, 6430, The Netherlands; 3 Department of Rehabilitation Medicine, Maastricht University Medical Center (MUMC+), P.O. Box 5800, Maastricht, AZ, 6202, The Netherlands; 4 Faculty of Psychology and Neurosciences (FPN), Department of Clinical Psychological Sciences (CPS), Maastricht University, P.O. Box 616, Maastricht, MD, 6200, The Netherlands

**Keywords:** Motivational interviewing, Motivation, Adherence, Design article, Drop-outs, Working mechanism, Mediators/moderators, Societal participation

## Abstract

**Background:**

Non-adherence and drop-out are major problems in pain rehabilitation. For patients with various health problems, motivational interviewing (MI) has shown promising effects to tackle these problems. In chronic pain patients, the effectiveness of MI is however unknown. Therefore, a MI-based pre-pain rehabilitation intervention (MIP) addressing motivation, expectations, and beliefs has been developed to prepare eligible patients for rehabilitation treatment.

**Methods/design:**

Study design: A parallel randomized controlled trial including two interventions: a motivational interviewing pre-pain rehabilitation intervention (MIP) and a usual care (UC) control arm. Follow-up will be 6 months after completion of rehabilitation treatment.

Study population: One hundred and sixty (*n* = 80 per arm) patients with chronic non-specific musculoskeletal pain visiting an outpatient rehabilitation department, who are eligible to participate in an outpatient cognitive behavioral pain rehabilitation program.

Intervention: MIP consists of two sessions to prepare and motivate the patient for pain rehabilitation treatment and its bio psychosocial approach. UC consists of information and education about the etiology and the general rehabilitation approach of chronic pain. Both the MIP and UC contain two sessions of 45 to 60 minutes each.

Objective: The aim of the current study is to evaluate the effectiveness of MIP compared to UC in terms of an increase in the long-term level of societal participation and decrease of drop-out during rehabilitation treatment.

Main study endpoints: Primary outcome is the change in level of participation (according to the ICF-definition: ‘involvement in a life situation’) 6 months after completion of rehabilitation treatment. Secondary outcomes are adherence and treatment drop-out, disability, pain intensity, self-reported main complaints, (pain-specific) self-efficacy, motivation, and quality of life. Costs are calculated including the costs of the pre-treatment intervention, productivity losses, and healthcare utilization. Potential moderators and active ingredients of MI are explored. For the process evaluation, parameters such as MI fidelity, feasibility, and experiences are explored.

**Discussion:**

The results of this study will provide evidence on the effectiveness of this MI-based pre-treatment in pain rehabilitation. Furthermore, a cost-effectiveness analysis and exploration of moderating and working mechanisms of MI and an extensive process evaluation takes place.

**Trial registration:**

Nederlands trial register NTR3065

## Background

Chronic non-specific musculoskeletal pain is a major health burden. It occurs in approximately 10% of the general population [1] and causes disability [2], medical expenses [3], and a high amount of work absenteeism [4].

In the Netherlands, cognitive behavioral therapy (CBT)-based approaches are part of usual rehabilitation care for patients with non-specific chronic pain [5], and also recommended in the Dutch guidelines [6]. The common assumption of CBT in rehabilitation is that pain and disability are not only influenced by biomedical factors but also by psychological and social factors, which is referred to as the bio psychosocial approach [7]. The primary aim of rehabilitation treatment is to increase the patient’s ability to cope with pain instead of curing pain. The ultimate intention is to increase the patient’s level of participation in society and his/her quality of life [8]. The ultimate goal and key outcome of rehabilitation is therefore societal participation. According to the International Classification of Functioning, Disability and Health (ICF), participation is therein defined as ‘involvement in a life situation’ whereas the opposite, participation restrictions, are defined as ‘problems an individual may experience in involvement in life situations’ [9].

The Initiative on Methods, Measurements, and Pain Assessment in Clinical Trials (IMMPACT) does not mention the measurement of participation [10]. But in the field of rehabilitation, participation is seen as an important concept in order to reflect meaningful patient-centered outcomes [11]. It is suggested that those outcomes should be included more often in trials [12].

Reviews have shown a moderate effectiveness of cognitive behavioral treatments in chronic non-specific musculoskeletal pain [13] and low back pain [14]. Behavioral therapy is more effective than usual care in terms of pain relief [15], and similar effects are shown for functioning after an intensive multidisciplinary bio-psycho-social rehabilitation program [16].

Unfortunately, in the current rehabilitation care, non-adherence and drop-out are major problems. Adherence rates are low in patients with chronic conditions [17], and subsequently, drop-out of pain rehabilitation programmes, ranging from 9% to 42%, is high [18-20]. Previous research showed that adherence and non-drop-out of treatment is related to a better outcome in physical and emotional functioning and pain severity [21]. Adherence is influenced by multiple factors such as the healthcare provider-patient relationship, the patient’s self-efficacy to be able to make changes [22], and the patient’s satisfaction with improvement [23].

In order to improve adherence and motivation to prevent drop-out, and to strengthen self-efficacy, motivational interviewing (MI) has been proposed [24]. Miller and Rollnick, the founders of MI, defined it as ‘directive, client-centred counselling style for eliciting behaviour change by helping clients to explore and resolve ambivalence’ [25]. The overall goal is to increase the client’s intrinsic motivation to change and enhance behavioral change [26]. MI was originally developed for problem drinkers [27] and in the beginning applied in the addiction field only (for example, [28,29]). Meta-analyses showed that using MI as a pre-treatment yielded the best outcomes compared with other active treatments [30-32]. Therein, MI was designed to prepare clients for further treatment such as CBT or an inpatient program. Furthermore, the effective application ranged from a variety of disorders such as addiction (except smoking cessation), increasing healthy behaviors, and the management of chronic illnesses. MI as treatment approach is fairly new in the field of chronic pain. MI has been applied with promising results in two small randomized controlled trials (RCTs) within primary care for patients with chronic pain conditions. Habib *et al*. [33] found significant increases in attending self-management workshops after a psychologist-led two-session MI-based feedback interview compared with an attention placebo interview [33]. Another recent study found a MI-adapted intervention added to outpatient physiotherapy for patients with back pain effective in enhancing motivation and exercise adherence compared to physiotherapy alone [34].

With the results of these studies in mind, it is seems promising to explore the effects of MI on non-adherence and drop-out in chronic pain rehabilitation care, and participation afterwards, where patients are characterized by a high levels of disability and complex problems.

### Aims

The primary objective of this current project is to study the effectiveness of MI by means of the MIP intervention. The interventions aims at decreasing drop-out rate and increasing adherence to treatment program to reach a high level of societal participation ultimately in patients with non-specific musculoskeletal chronic pain, who have been selected for pain rehabilitation treatment.

The main research questions are:

1. What is the effectiveness of MI-based pre-treatment (MIP) compared to usual care (UC) on the level of participation, treatment drop-out rate, and adherence in patients with chronic non-specific musculoskeletal pain following pain rehabilitation?

2. What is the cost-effectiveness and cost-utility of MIP, compared to UC from a societal perspective?

3. What is the feasibility of the MIP intervention in terms of MI fidelity (process evaluation)?

4. What are experiences of nurses and patients in terms of satisfaction with and barriers of the MIP intervention (process evaluation)?

### Hypotheses

1a. It is expected that patients’ level of social participation will be higher in the MIP intervention condition compared to the UC after pain rehabilitation treatment and at 6-month follow-up after finishing the rehabilitation treatment.

1b. It is hypothesized that drop-out rates will be lower and adherence to the treatment and the level performing daily activities) will be higher in the MIP condition compared to the UC.

2. It is hypothesized that MIP will be more efficient compared to UC, both in effects as well as utilities.

3. It is hypothesized that the MIP intervention is feasible for the participating nurses and the MI fidelity of the MIP intervention is sufficient.

## Methods/Design

### Study design

The PREPARE study is a parallel single-blind RCT.

### Recruitment

Participants will be recruited from the outpatient department of Rehabilitation Medicine in an academic and a regional hospital in the Southern part of the Netherlands, starting in January 2012 and lasting till June 2013.

Patients visiting one of the participating outpatient rehabilitation departments for an intake interview will be evaluated whether they are eligible for rehabilitation treatment by the consultant in rehabilitation medicine.

### Participants

Participants are patients who meet the inclusion criteria as stated below.

In current care, patients are selected for rehabilitation treatment by the consultant in rehabilitation medicine based on expert opinion. For this, both medical (origin and severity of the pain problem and interfering co-morbidity) and motivational factors are evaluated. In patients eligible for outpatient pain rehabilitation treatment, additional inclusion and exclusion criteria for participation in the PREPARE study are checked and in case of eligibility, patients will be invited to participate.

Inclusion criteria are: non-specific chronic (duration >3 months) musculoskeletal pain; age between 18 and 65 years; the chronic pain syndrome is not attributable to a recognizable, known specific pathology (for example, infection, tumor, osteoporosis, fracture, structural deformity, inflammatory disorder (for example, ankylosing spondylitis); medium to high level of motivation for pain rehabilitation from the consultant’s perspective; adequate literacy to complete assessment measures.

Exclusion criteria: pregnancy; surgery planned in the foreseeable future; patient involved in litigation procedures; suspicion of a psychiatric disease that will interfere with rehabilitation treatment (according the expert opinion of the consultant in rehabilitation medicine).

### Sample size

The sample size calculation is based upon an estimated difference in change in level of participation between baseline and 6-month follow-up (after completion of the rehabilitation treatment) as assessed by the Utrecht Scale for Evaluation of Rehabilitation-Participation (USER-P) [35,36]. Reproducibility [37], responsiveness [38], and validity [37] of the USER-P are satisfactory. As participation is the important outcome in rehabilitation, we have chosen this as primary outcome. The scale consists of the three aspects of participation represented by the subscales Frequency, Restriction, and Satisfaction. The sum score of each subscale is converted to scores on a scale ranging from 0 to 100. There is no USER-P total score.

Our sample-size calculation is based upon the subscale Satisfaction. This subscale is the most important part of participation as it marks the subjective experience of participation [9]; therefore we have chosen this one.

As at this moment no information is available about the USER-P in a pain population regarding normative values, clinically relevant change, and clinically relevant difference, our study is exploratory of character. In a study by van der Zee (2011) the USER-P is taken in different patient groups at three different points in time [38]. As these groups were part of a cohort study, nothing is known about differences after a specific treatment. We know from other studies that multidisciplinary pain rehabilitation treatment is moderately effective with effect sizes ranging from 0.30 for behavioral outcomes up to 0.40 for functional outcomes [14]. We expect an additional effect for the MIP intervention since systematic reviews of motivational interviewing-based interventions show an effect size ranging from 0.27 up to 0.40 when compared with a weak comparison group [30-32,39]. It has to be said that in those reviews no study in the field of chronic pain or rehabilitation treatment was taken into account. Therefore, our power calculation is based on the following two assumptions:

1. Using an expected mean change of 5 points within UC is expected whereas a difference of additional 5 points (thus at least 10 points in total) within MIP in the USER-P sub scale Satisfaction is considered as clinically relevant. Those numbers are estimates as the clinically relevant difference is not known yet.

2. Assuming a standard deviation of 10 points in both groups, the standardized effect size, Cohen’s d, is (10–5)/10 = 0.5 indicating a medium additional effect size of the MIP [40].

Assuming power (1-β) of 80%, α = 0.05, two-sided testing, and holding the above mentioned assumptions, would necessitate *n* = 128 participants (*n* = 64 per arm). With an expected total drop-out of 20% (10% drop-out from the study, another 10% drop-out during the usual rehabilitation treatment as our unsystematic registration has shown), *n* = 160 (*n* = 80 per arm) was chosen as the optimal total sample size. Additionally, as the recruitment of 160 participants in the region of South-Limburg/the Netherlands is feasible in a time range of 18 months, we have chosen for this amount of participants.

### Informed consent, randomization, and blinding

Standard informed consent procedures will be used. After having received informed consent, the participants are randomly assigned to either the experimental intervention condition (MIP) or the control condition (UC). Block randomization with block size of four are used per study site (hospital) to obtain equal numbers in both arms. An independent research assistant will execute the randomization by means of a computerized random number generator resulting in a computer-generated list of random numbers to allocate the participant either to the experimental or the control condition. The consultant in rehabilitation medicine, the rehabilitation team and the participant will all be blind for the result of the allocation to the UC or the MIP but not for participation in the study at all. The research assistant responsible for the logistics of the present study and the participating nurses administering the intervention and control condition are not part of the treatment team and are not blinded for allocation.

### Treatment

To understand the features of both PREPARE conditions, first current care in pain rehabilitation in the Netherlands will be explained. Thereafter, both PREPARE conditions are described. At last, the role of the nurses is illustrated.

#### Current care in pain rehabilitation

In the current pain rehabilitation treatment in the Netherlands [41] roughly four phases can be identified. The specific content of the phase can differ per center.

The four phases are:

1. Screening: In this phase, a patient’s eligibility and readiness for pain rehabilitation is assessed. Also information is provided by either a consultant in rehabilitation medicine or a nurse practitioner. In most centers for this purpose use existing educational material, such as ‘De pijn de baas’ (‘Mastering pain’) [42] or ‘Explain Pain’ [43] (WPN, 2012, internal document) are used as information tools for this purpose. In most places, information is given before the definite start of the treatment.

2. Observation: A patient’s pre-treatment situation and motivation is assessed, and the most suitable type of treatment is selected. In some centers, this phase takes two or three sessions in 2 to 4 weeks. Often this is accompanied by the use of self-report questionnaires.

3. Rehabilitation treatment: The multidisciplinary pain rehabilitation treatment lasts for around 3 months with an intensity of 2 or 3 days a week on average. Depending on the patients’ limitations and underlying problems, different treatment approaches such as graded activity and/or graded exposure are used in combination with other psychosocial and physical interventions.

4. Aftercare and post rehabilitation treatment assessment: Booster sessions, tailored to patients’ needs, are given for relapse prevention and long-term facilitation of applying learned skills to maintain and further improve participation. The moment of the assessment is around 6, 12, 24, to 52 weeks post-treatment. These follow-up appointments are mostly accompanied by completion of self-report questionnaires for treatment evaluation. It has to be said that this is not routine care in all Dutch rehabilitation centers.

#### PREPARE conditions

**Figure 1:**
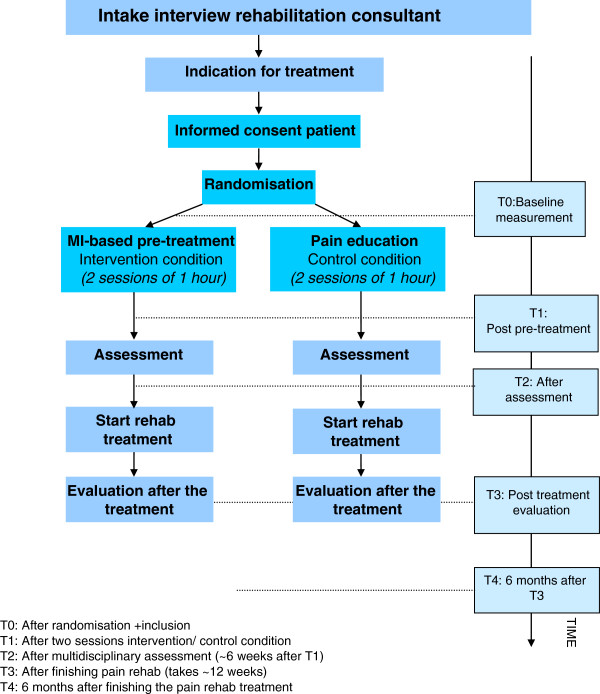
Flow-chart of the design of the PREPARE study (apart file).

The PREPARE intervention focuses on phase one. This is also the period during which patients are waiting before they can start their actual treatment (observation and rehabilitation treatment). During the study, patients will receive either the new MI-based intervention or care as usual. In order to standardize treatment options, both arms (MIP as well as UC) will include two sessions with identical contact time. In order to prevent contamination of treatment, each treatment condition will be delivered by different nurses. Figure 1 illustrates the situation during the PREPARE study.

1. Usual care (UC)

In the usual care (UC) arm, participants will receive pain education according to the information ‘De pijn de baas’ [40] (Mastering pain). The ultimate goal is to provide the participant with information about, for example, differences between acute and chronic pain, its nature, and strategies to handle cognitions about pain.

2. MIP intervention condition

The MI intervention condition is based on the four general principles of MI, which are incorporated into all sessions in the MIP condition [44]:

1. Expressing empathy by the use of reflective listening,

2. Developing discrepancy between client goals and current problem behavior,

3. Rolling with resistance by avoiding argumentation by assuming that the client is responsible for the decision to change,

4. Supporting self-efficacy and optimism for change [44].

The features of both MIP and UC are mentioned in Table 1.

**Table 1:** Features of the two interventions

**Motivational interviewing based pre-treatment (MIP)**	**Pain education (Usual care (UC))**	
Goal: explore participant’s life situation, impairments and ambivalences in order to enhance intrinsic motivation	Goal: provide the participant with information	Goal
General principles of MI	General principles of health education and patient education	Basic principle
Content based on patient-driven topics derived from the ICF-model	Content based on the book ‘De pijn de baas’ [42] (Mastering pain)	Foundation
Content sessions tailored to the patients’ readiness to change	Content is fixed by means of the treatment protocol	Protocol rigidity
Exploration actual (life) situation, burden and impairments of the chronic pain in daily life	Provision of general health education about topics related to chronic pain	**Content first session**
Assessing + enhancing motivation, self-efficacy, and readiness to change for behavior	Provision of information regarding core elements of pain rehabilitation	
Giving feedback adapted to the state of readiness-to-change	Continuation of general health education about topics related to chronic pain	**Content second session**
Continuation exploration		
Continuation assessing + enhancing motivation, self-efficacy, and readiness to change for behavior		
Is given related to the stage of change of the participant according to the MI-principles	Is not given	Feedback

The nurse is directive within this process, but the participants’ autonomy is strengthened and his or her right to decide on everything related to the person is respected.

During the first appointment, a trustful relationship between participant and nurse is built, the actual (life) situation, burden, and impairments of the chronic pain in daily life, motivation, self-efficacy, and readiness to change behavior is assessed and enhanced. Next, the session is summarized and closed. The second appointment starts with a brief motivational feedback session. The process of the first appointment will be discussed with the participant by giving feedback adapted to the state of readiness-to-change. Therein, motivation and self-efficacy for behavior change is enhanced. In addition, topics related to chronic pain and treatment, such as education about the influence of exercise and a background in the bio psychosocial approach will be discussed. Next, the session is summarized and closed.

#### Nurses

Both UC and MIP are administered by registered nurses working in the field of rehabilitation. In order to prevent contamination of treatment by components of the other intervention, nurses will be trained and only guide participants in one specific condition. The two nurses selected to deliver the MI condition, are both experienced MI coaches (4 years of experience). To assure optimal quality of the MI condition, both nurses receive an expert training by a certified MI trainer: their MI knowledge and experience in the context of chronic pain rehabilitation are updated based on an evidence-based training tailored to their specific needs [45-49]. During the study the MI sessions will also be audio taped and the MI quality will be assessed using a coding instrument (Motivational Interviewing Treatment Integrity (MITI)) (see also Methods section, Process evaluation section). Subsequent training will be based on additionally regular supervision, done by training on the job and direct feedback on MIP sessions.

Quality of the UC condition:

The two nurses selected to deliver the usual care condition are experienced in the field of rehabilitation (20 years and 5 years, respectively).

Before the start of the study, they will receive a 3-h refresher training in general communication skills and general principles of health education. In addition, the content of relevant chapters of the book ‘Mastering pain’ is discussed. During the study, the nurses of the UC condition will meet once or twice a year to discuss problems encountered during the sessions.

### Data collection

For the (cost-) effectiveness evaluation, measurements will be carried out before the first MIP/UC pre-treatment sessions (T0), after the MIP/UC (T1 = post pre-treatment), after the regular multidisciplinary pre-rehabilitation assessment (before starting rehabilitation), at the start of the pain rehabilitation treatment (T2), after treatment completion (T3), and at 6-month follow-up (T4). Study assessments at T0, T2, and T3 will be integrated in current care clinical assessment battery pre-rehabilitation and post rehabilitation (see also Figure 1 (flow-chart) and Table 2). Cost measurements will be carried out at three points: T0, T2, and T4. Information on drop-out from and adherence to the (pre-) rehabilitation treatment, start and end date of the treatment, and no show are derived from the administrative patient registry.

**Table 2:** Outcome measures, abbreviation instrument, and assessment moments PREPARE study

		**Baseline**	**Two sessions**	**After pre-treatment**	**Start pain rehab treatment**	**Finish pain rehab treatment**	**6 months after T3**
	**Abbr.**	**T0**^**a**^		**T1**	**T2**^**a**^	**T3**^**a**^	**T4**
**Covariates**							
Demographics patient		x					
***Primary outcome effect evaluation***							
Participation	USER-P	x		x		x	x
***Secondary outcomes effect evaluation***							
Physical functioning/disability	PDI	x			x	x	x
Pain intensity	VAS	x			x	x	x
Main complaints	PSC	x			x	x	x
Drop-out/adherence				#		#	
Self-efficacy/perceived competence	GSE	x		x		x	x
Pain-specific self-efficacy scale	CPSS_V2	x		x		x	x
***Cost-effectiveness evaluation***							
Cost questionnaire	Tic-P	x			x		x
Productivity losses	SF-HLQ	x			x		x
Quality of life/health status	SF-36	x			x	x	x
**Potential active ingredients MI**							
Stages of change	MPRCQ 2	x		x			
MI integrity/fidelity			*				
**Potential moderators**							
Credibility and expectancy of the treatment	CEQ	x		x	x		
Motivation	TMQ	x		x			
Depression	BDI	x			x	x	
Acceptance	AAQ-II	x		x		x	
Flexible goal-adjustment	FGA	x		x		x	
**Process evaluation**							
Satisfaction with (pre-)treatment				x +			
Client-centeredness	CCCQ			x			
Feasibility				+			

x Questionnaires in patients.

+ Questionnaire in nurses.

# Process measures.

* Audiotapes.

^a^Alongside usual care assessment.

As patients have to incorporate learned behavior into daily routine functioning, the improvement of participation, the ultimate goal of pain rehabilitation, is not optimal directly after treatment. We expect relevant change in the level of participation to occur at 6 months. Therefore this time point is chosen as our last measurement moment. Due to the fact that the PREPARE trial is embedded in clinical practice, the measurement moments are not precisely planned at the same moment. Especially the waiting list period between T1 and T2 may vary and furthermore the total duration of the pain rehabilitation treatment may vary between 10 to 16 weeks.

### Demographic and medical variables

Baseline assessment includes: gender, nationality, marital status, educational level, and co-morbidity, as well as pain location and pain duration.

In addition, baseline assessment includes the following risk factors for attrition: sick leave [50], level of being active in sports, smoking status [18], level of physical functioning [50], pain intensity [50,51], perceived disability [51], low treatment satisfaction [52], and expectations regarding the content of the treatment [53].

### Outcome measures

Outcome measures (parameters) and assessment moments are presented in Table 2.

#### Primary outcome

The primary outcome of the effect-evaluation will be the mean difference in change in level of participation of the participants at T4 compared to T0 (baseline).

Participation will be measured by the Utrecht Scale for Evaluation of Rehabilitation-Participation (USER-P) [54]. It consists of 32 items and covers three aspects of participation, namely Frequency, Restriction, and Satisfaction. The subscale Frequency consists of 12 items and assesses vocational activities, and frequency of leisure activities and social activities. Each item is scores from 0 (‘not at all’) up to 5 (‘36 hours or more’/’19 times or more’). The subscale Restriction comprises 10 items assessing experiences in vocational and leisure activities. Items are rated on a four-point Likert scale ranging from 0 (‘not possible at all’) to 3 (‘independent without disability’). The subscale Satisfaction consists of 10 items and rates satisfaction regarding participation in daily life issues such as work and social relations. Items are rated on a five-point Likert scale ranging from 0 (‘not satisfied at all’) to 4 (‘very satisfied’). Higher scores reflect more social participation (each higher frequency, less restrictions, higher satisfaction). The psychometric qualities validity and reproducibility are satisfactory [36,55].

#### Secondary outcomes

Level of performing daily activities resulting from chronic pain will be measured by the Pain Disability Index (PDI) [56], which consists of seven items using an 11-point Likert scale ranging from 0 (no disability) to 10 (total disability). A total score is derived by summing up the item responses, ranging from 0 up to 70, with a higher score indicating more disability [56]. Good psychometric properties of the PDI have been shown and normative data are available [57].

Pain intensity is assessed by a 100-mm Visual Analogue Scale (VAS). The VAS is a common and valid tool for measuring pain intensity [58].

In order to assess the participant’s self-reported main complaints, the Patient Specific Complaints questionnaire (PSC) will be used [59,60]. The participant selects three to five most limited functional activities and rates the difficulty to perform them during the previous week on a 100-mm VAS [60]. It is tested as valid, reliable, and responsive in patients with chronic musculoskeletal pain [61,62].

Drop-out will be registered in the patient registry in the institution. Each patient will be classified as either a participant who has completed the pain rehabilitation program as proposed or who has dropped out prematurely. In addition, the attendance of each treatment session will be assessed. The level of adherence to the pre-treatment intervention (MIP and UC) is computed by dividing the number of the PREPARE sessions that an individual participant actually has attended by the number of two pre-treatment sessions as planned.

Adherence to the rehabilitation treatment is computed by dividing the number of scheduled treatment sessions (which amount is tailored to the participants’ needs) by the actual attended number of visits. This method has been reported extensively in the literature as a measure of adherence to rehabilitation [63].

General self-efficacy is assessed by the Dutch General Self-efficacy questionnaire [64]. Ten items ranging from ‘totally untrue’ up to ‘totally true’ assess coping in general life demands. A confirmatory factors analysis has taken place in elderly [65] showing a good fit.

A disease-specific self-efficacy measure is used, namely the Chronic Pain Self-efficacy Scale (CPSS) consisting of 22 items the three subscales pain management, coping, and physical function. It offers good overall internal consistency and acceptable test-retest reliability [66].

#### Cost-effectiveness and cost-utility

To evaluate the economic effects of MIP and UC, relevant cost categories of resource use and volumes of these categories will be measured. Finally, volumes are multiplied by the corresponding costs.

The direct (non-)healthcare costs will be assessed with the Trimbos/iMTA questionnaire for Costs associated with Psychiatric Illness (Tic-P) [67]. This self-reporting questionnaire consists of 15 items and assesses healthcare use in a recall period of 3 months.

Productivity losses are assessed by the Short Form Health and Labor Questionnaire (SF-HLQ) [68]. The SF-HLQ measures the extent of production losses of paid and unpaid work in four modules: absence from work, reduced productivity at paid work, unpaid labor production, and impediments to paid and unpaid labor. It consists of 11 items.

For the cost valuation, standardized cost prices will be used from the Dutch manual for cost analysis in healthcare research [69]. Where no standard cost prices are available, real costs or tariffs will be used to estimate costs. Productivity losses will be calculated based on the friction costs method. Cost prices will be presented in Euros and the baseline year is 2012, or otherwise discounted. The discounting rate of costs is 4%, and 1.5% for effects [70]. To analyze differences in costs, costs per patient-year will be calculated.

For the cost-effectiveness analysis (CEA), costs will be weighted against the primary outcome measure participation. The measure is described above.

For the cost-utility analysis (CUA), costs per year will be weighed against utilities based on the SF-36. The SF-36 is a reliable and valid instrument to measure health-related quality of life [71]. Utilities are values or preferences that respondents assign to a particular health state and are overall expressed on a scale ranging from 0 to 1 [72]. The utilities used in this study will be derived with an algorithm of the SF-6D, which estimates utilities based on the health-related quality of life scores of the SF-36 [73]. Furthermore, the utilities will be estimated by an algorithm of Brazier, Roberts, and Deverill (2002). The derived utilities at three measurement points (T0, T2, T4) will be used to compute the Quality Adjusted Life Years (QALY) score by means of the area under the curve method [74]. This means that the weights for each health state (utilities) will be multiplied by the time in that particular health state and then summed to calculate the total number of QALYs.

#### MI-specific possible active ingredients

Resulting from the existing literature on MI, potential specific active ingredients were identified and will be assessed in this study [75,76].

The stage of change is assessed by the Multidimensional Pain Readiness to Change Questionnaire 2 (short version). It measures the chronic pain participants’ readiness to change. The questionnaire is based upon the trans theoretical model of behavior change of Prochaska and DiClemente. The MPRCQ2-26 consists of 26 items and is scored on a seven-point Likert scale [77]. Psychometric properties are evaluated and appeared to be satisfactory [78]. To be able to take it into account as possible moderator, it is measured before and after the pre-treatment.

MI integrity is hypothesized to be another working mechanism for MI. It is further explained below under the heading ‘MI integrity and fidelity’.

#### Possible moderators

We will also measure specific individual factors at baseline that could influence the treatment effect. From the literature, a number of psychosocial attributes moderating the effects of the pain rehabilitation treatment are identified [79].

Research has shown the influential role of expectancy on treatment outcome in pain rehabilitation [80,81]. Credibility and expectancy is measured by an adapted and translated version of the Credibility and Expectancy Questionnaire (CEQ) [82]. In two parts, participants are asked to rate in total five items related to the credibility and expectancy regarding pain rehabilitation treatment. Credibility and expectancies regarding the treatment have to be rated in two items. In another item the expected success in terms of an improvement in participation, decrease in disability, and decrease in pain intensity are rated. The CEQ uses a rating scale, ranging from 1 (not at all) to 9 (very much). Good psychometric properties have been demonstrated and the same factors structure was confirmed [82].

Motivation is assessed by the Treatment Motivation Questionnaire (TMQ). The TMQ assesses intrinsic and extrinsic information about entering and remaining in treatment [83]. It consists of 26 items to be rated on a five-point Likert scale ranging from 1 (not at all true) up to 5 (very much true). The factors internal and external motivation, interpersonal help seeking and confidence in treatment are represented. Items are slightly adapted to the rehabilitation context. The TMQ correlates well with professionals’ rating of the abovementioned factors what suggests good construct validity [83].

Depressive symptoms are hypothesized to moderate the effects of the rehabilitation outcomes. Therefore, the level of depression is assessed by the Becks Depression Inventory (BDI) [84]. The BDI is a well-known instrument, and suitable for its use in pain research. It is a reliable, valid, and widely-used instrument [85,86].

Acceptance has shown to explain a significant amount of variance in the prediction of patient functioning and suffering from chronic pain [87]. Acceptance is assessed by the 10-item Acceptance and Action Questionnaire-II (AAQ-II) [88] and scored on seven-point Likert scale items ranging from ‘never be true’ up to ‘always true’. The Dutch version has shown a high internal consistency and a good validity [89].

Flexible goal-adjustment (FGA) has shown to be a moderator in the relation between self-discrepancies and negative emotions [90]; flexibility has shown to be of moderating influence on pain willingness and activity engagement [91]. FGA, a component of psychological flexibility is measured with the Tenacious Goal Pursuit and Flexible Goal Adjustment Scale Brandstädter and Renner Questionnaire [92], which assesses the tendency to adjust personal goals and standards to situational limitations. It consists of 15 items with five possible answers ranging from ‘totally agree’ up to ‘not agree at all’. The scale’s internal consistency is satisfactory (α = 0.80) [92].

#### Process evaluation: MI integrity and MI fidelity

To check whether MI was implemented and delivered as intended, an evaluation of treatment fidelity and treatment integrity is important.

Also given the explicit emphasis in MI as a spirit rather than a technique [93], treatment fidelity and treatment integrity will be measured. Therefore, the MI Treatment Integrity Code (MITI version 3.1) [94] will be used. It has shown to be a cost-effective and reliable tool [95,96], which is also validated to check MI fidelity [94,97]. Feedback during the training and during intervision sessions will be delivered based also on the MITI. Research has also shown that proficiency rating by skilled coders predicted treatment outcome [98-100]. To be able to do so, all pre-treatment sessions are audio taped, and a random sample of 20% of all sessions will be scored by one of the authors (VCM). Twenty per cent out of those will also be scored by another skilled coder (NN) using the abovementioned MITI instrument.

#### Process evaluation in participants and nurses

A process evaluation is needed to evaluate the actual delivery of our pre-treatment intervention, the process evaluation focuses on the level of nurses and patients [101].

1. Patients: Patients will be asked to score their satisfaction with pre-treatment in a set of questions for process evaluation purposes at the end of the pre-treatment intervention (included in the questionnaire of T1). In case of treatment drop-out, a patient is asked to answer the questions regarding satisfaction with the (pre-) treatment directly after the decision to stop, including reasons for drop-out from treatment.

Furthermore, client-centeredness will be assessed by the Client Centred Care Questionnaire (CCCQ). The questionnaire was originally developed for the use in clients receiving homecare [102]. By making some minor revisions, it can also be used in the rehabilitation setting [103]. It has shown good psychometric qualities [102].

2. Nurses: After both pre-treatment sessions, nurses are asked to fill in a short questionnaire. Questions are asked about the steps taken during the intervention, the content of the discussion during the sessions, and the client’s active participation. The questionnaire’s structure is based on the work of Steckler and Linnan [101] and adapted to the specific situation of our study.

### Statistical analysis

The statistical analysis for the effect evaluation, the cost-effectiveness evaluation and cost-utility analysis, and the process evaluation will be described separately. Data will be analyzed using SPSS version 17.

#### Effect evaluation

Baseline data will be analyzed to describe the characteristics of all participants and to check for significant imbalances between the two groups. Possible baseline differences between the intervention group and the control group will be tested by an independent samples *t*-test (normal distribution) or Mann–Whitney U tests (non-normal distribution) in the case of continuous variables. In the case of dichotomous variables, a Chi-square test will be used. In case of imbalances in prognostic factors between both randomized groups, an adjustment for those factors will take place. The number of drop-outs and follow-up losses in both groups will be reported based on descriptive data. Differences in primary and secondary outcome measures between MIP and UC will be analyzed using repeated measurements techniques and multilevel analysis. This approach is chosen because repeated measures within individual subjects are taken. A *P* value <0.05 is considered statistically significant. Data will be analyzed according to the intention-to-treat principle. All outcomes will be assessed at each follow-up moment by taking into account a specific a priori hypothesis. Additionally, per protocol analyses will be performed to assess whether protocol deviations have caused bias.

### Moderation analysis

As secondary analyses, the working mechanisms of MI are investigated. For testing moderation, an interaction term of the potential moderator is constructed and analyzed by linear regression.

### Economic evaluation

In the economic evaluation, cost and effects of usual care and MIP will be calculated and compared. Therefore, the cost per patient year (=participant year) will be calculated. This means that all observed costs will be extrapolated to a 1-year period.

The analysis will include those persons for whom at least two of the three follow-up measurements (cost diaries) are available.

For the cost-effectiveness analysis (CEA) the cost-effectiveness ratio will be stated in terms of costs per improvement on participation (as assessed with the USER-P).

For the cost-utility analysis (CUA) the cost-utility ration will be stated based on the cost per (Quality Adjusted Life Year) QALY gained.

Bootstrap (5,000 times) re-sampling techniques will be used to test for differences and uncertainty in costs and effects between the MIP and the UC intervention.

The bootstrapped cost-effectiveness and cost-utility ratios will be subsequently plotted in a cost-effectiveness and cost-utility plane. The results of this study will also be depicted in cost-effectiveness acceptability curves (CEACs) [104].

### Process evaluation

Results of the questionnaires for the process evaluation of participants and nurses will be analyzed descriptively. A summary score of MI fidelity will be computed by ratings of the MI-consistent behavior evaluated by the MITI. Summary score thresholds are known for beginning proficiency level and competency level [94]. A binary variable MI fidelity will be computed and used in analyses for the effectiveness of MI as well as working mechanisms of MI. MI fidelity is furthermore used in mediation analyses as hypothesized mediator in the path between the MI intervention and its effect.

## Discussion

During the design of this study many choices and selections were made, and three of the most crucial ones will be discussed.

First, we specifically chose for participation as our primary outcome measure instead of for example disability. This is in accordance with the main outcome as suggested in the ICF. The ICF is nowadays accepted in most countries to describe and measure health and disability [105], and the relevant aim of rehabilitation. Therefore, it is pre-eminently adequate to use participation as primary outcome measure in the field of rehabilitation [106]. The choice of the other current outcome measures is congruent with the IMMPACT recommendations [10,107,108].

Second, we chose for an attention control group instead of a no-treatment group. Reasons for our choice are: As we hypothesize that attention of health providers alone could be a factor associated with motivation for treatment, it seems important to standardize for this factor when studying the effect of the content of a new intervention aiming at changing motivation for treatment. Therefore, we preferred the design of a trial comparing a MI-based and an attention control pain education above the design of a trial with a single intervention and one waiting list condition without control intervention. Since the content of pain education is part of usual care in the Netherlands, we designed an attention control intervention based on the content of usual care. For comparability reasons, the duration of the attention control condition is identical to the MI intervention.

The quality and the efficacy of MI delivery are troublesome within some research reports, and the documentation of MI fidelity is often lacking (for example, [34,109]). Therefore, we lay a strong emphasis on the MI-training of the care providers and an ongoing MI-fidelity check. MI training is provided in order to make nurses adequately proficient to learn the principles and acquire the skills of MI [46,99,110,111]. MI integrity is checked alongside the trial for two aims: For feedback about MI proficiency during intervision with the nurses [95] and for the interpretation of MI effects since intervention fidelity is an important prerequisite for intervention effects.

To conclude, this paper describes the design of a randomized controlled trial to study the effectiveness of a nurse-led motivational interviewing-based pre-treatment intervention aimed at improving and sustaining participation up until 6 months after the pain rehabilitation. The results of this study will provide evidence of the effectiveness of this pre-treatment intervention as well as insights in working mechanisms as well as cost-effectiveness and cost-utility and moderating mechanisms of such an intervention in a chronic pain population.

## Ethical approval

The study protocol was approved by the Medical Ethical Committee of University Hospital Maastricht and Maastricht University. The study is registered in a public trial registry (Nederlands Trial Register NTR3065).

### Trial status

PREPARE is an ongoing trial and it is expected that patient recruitment will last till approximately June 2013.

## Abbreviations

MI: Motivational interviewing; MIP: Motivational interviewing-based pre-treatment; Prepare: Pre-Pain rehabilitation; UC: Usual care.

## Competing interests

This PhD project is funded by Adelante zorggroep, Hoensbroek, The Netherlands.

## Authors’ contributions

VCM, MG, JV, AK, and RS participated in the concept, design of the study, and research protocol. All authors read and corrected draft versions of the manuscript and approved the final manuscript.
